# Cementless short-stem total hip arthroplasty in the elderly patient - is it a safe option?: a prospective multicentre observational study

**DOI:** 10.1186/s12877-019-1123-1

**Published:** 2019-04-17

**Authors:** Georgios Gkagkalis, Patrick Goetti, Sabine Mai, Ingmar Meinecke, Näder Helmy, Dominique Bosson, Karl Philipp Kutzner

**Affiliations:** 1 Department of Orthopaedic Surgery, Hôpital du Sacré-Cœur, Université de Montréal, 5400 boul. Gouin Ouest, Montréal, QC H4J 1C5 Canada; 2 Department of Orthopaedic Surgery and Traumatology, Lausanne University Hospital – CHUV, Rue du Bugnon 21, 1011 Lausanne, Switzerland; 3 Vitos Orthopaedic Clinic Kassel, Wilhelmshöher Allee 345, 34131 Kassel, Germany; 4 Helios Park-Clinic Leipzig, Strümpellstr. 41, 04289 Leipzig, Germany; 5 Bürgerspital Solothurn, Schöngrünstr. 42, 4500 Solothurn, Switzerland; 6 Department of Orthopaedic Surgery, Nyon Hospital, Chemin Monastier 10, 1260 Nyon, Switzerland; 7 Department of Orthopaedic Surgery and Traumatology, St. Josefs Hospital Wiesbaden, Beethovenstr. 20, 65189 Wiesbaden, Germany

**Keywords:** Total hip arthroplasty, Short stem, Age, Elderly, Young, Optimys

## Abstract

**Background:**

Due to its bone preserving philosophy, short-stem total hip arthroplasty (THA) has primarily been recommended for young and active patients. However, there may be benefits for elderly patients given a less invasive operative technique due to the short curved implant design. The purpose of this study was to compare the clinical and radiological outcomes as well as perioperative complications of a calcar-guided short stem between a young (< 60 years) and a geriatric (> 75 years) population.

**Methods:**

Data were collected in a total of 5 centers, and 400 short-stems were included as part of a prospective multicentre observational study between 2010 and 2014 with a mean follow-up of 49.2 months. Preoperative femur morphology was analysed using the Dorr classification. Clinical and radiological outcomes were assessed in both groups as well as perioperative complications, rates and reasons for stem revision.

**Results:**

No differences were found for the mean visual analogue scale (VAS) values of rest pain, load pain, and satisfaction, whereas Harris Hip Score (HHS) was slightly better in the young group. Comparing both groups, none of the radiological parameters that were assessed (stress-shielding, cortical hypertrophy, radiolucency, osteolysis) reached differences of statistical significance. While in young patients aseptic loosening is the main cause of implant failure, in the elderly group particularly postoperative periprosthetic fractures due to accidental fall have to be considered to be of high risk. The incidence of periprosthetic fractures was found to be 0% in Dorr type A femurs, whereas in Dorr types B and C fractures occurred in 2.1 and 22.2% respectively.

**Conclusions:**

Advanced age alone is not necessarily to be considered as contra-indications for calcar-guided short-stem THA, although further follow-up is needed. However, markedly reduced bone quality with femur morphology of Dorr type C seems to be associated with increased risk for postoperative periprosthetic fractures, thus indication should be limited to Dorr types A and B.

**Trial registration:**

German Clinical Trials Register; DRKS00012634, 07.07.2017 (retrospectively registered).

## Background

Total hip arthroplasty (THA) has been deemed as “the operation of the century” due to its excellent clinical outcome and patient satisfaction rates [1]. This highly effective procedure was originally intended for elderly, low-demand patients but improvements in implant technology, tribology, and surgical techniques led to the extension of indications over a wide range of ages. Thus, nowadays THA is increasingly offered to young and active patients as well as to elderly and less demanding ones with end-stage osteoarthritis of the hip.

The use of cementless short stems in THA has been rising in parallel with the use of minimally invasive approaches [2–4]. Short stems have been developed in an effort to address various issues, such as bone preservation of the proximal femur, the reduction of stress shielding and mid-thigh pain incidence as well as to facilitate the use of soft tissue sparing procedures [5–7].

Due to its bone preserving philosophy, short-stem THA has initially been recommended for young and active patients with adequate bone quality [8]. Because of increased activity levels and longer life expectancy, these patients have a higher probability for revision during their life-span, and thus, bone preservation during the index procedure is crucial [9].

In geriatric populations, an overall reduced bone quality is more frequently observed, thus the usage of conventional stems with either cementless or cemented fixation is generally preferred [10, 11]. However, recent publications indicate that short stems may be an adequate treatment, providing rigid fixation, also for elderly patients [12, 13].

The use of certain conventional and thus more invasive stem designs has been shown to lead to an increased risk of intraoperative and postoperative femoral fractures [14]. A short-curved design of the stem could offer certain advantages for the patients, the elderly in particular, due to the less invasive operative technique. Molli et al. [15] proposed that a shorter, less broaching-demanding stem could decrease the incidence of intraoperative periprosthetic fracture compared to a standard-length stem, especially in the greater trochanter region, which is one of the most crucial causes for impaired postoperative function, especially in geriatric patients. Less blood loss and a lower transfusion rate have been reported with the use of short stems compared to straight stems [16]. Elderly patients could also benefit from shorter operative times [17]. In addition to that, cement-related complications remain a problem regarding mortality, especially in the elderly population in which additional comorbidities are more frequently encountered [18].

Achieving a good primary axial and rotational stability of the stem with a good metaphyseal fit and close cortical contact is very important in order to avoid subsidence and early aseptic loosening or periprosthetic fracture [19]. Sufficient primary stability and avoidance of proximal-distal mismatch are challenges to which a tapered short stem that provides metaphyseal anchorage may be a good solution [19, 20]. Kim et al. [21] showed that stable fixation and osseointegration might be achieved without any need of diaphyseal fixation in the young as well as in the elderly patients.

To date, very few results relating to new-generation short stems have been published for the geriatric population, and it remains unclear whether elderly patients benefit from the theoretical advantages of these designs.

The purpose of this study was to compare the clinical and radiological outcomes as well as perioperative complications of a cementless, metaphyseal engaging, calcar-guided short stem between a young (< 60 years) and a geriatric (> 75 years) population.

## Methods

Patients were included in 5 centers in Germany and Switzerland as part of a prospective multicentre observational study between 2010 and 2014. Ethical approval was obtained from the Freiburg Ethics Commission International (feki Code: 010/2071). The study conformed with the 1964 Declaration of Helsinki and its later amendments. Two age groups were created for analysis, the young group included patients younger than 60 years old, and the elderly group included those older than 75 years (Table 1). The study excluded all patients between 61 and 74 years of age, resulting in a total of 400 hips in 360 patients. The indications were primary hip osteoarthritis (69.5%), secondary hip osteoarthritis due to posttraumatic sequelae (11.2%), hip dysplasia (10.3%), osteonecrosis of the femoral head (7.2%) or other inflammatory arthritis (1.5%) and femoral neck fracture due to trauma (0.3%).

**Table 1:** Demographics of the two study groups

	Young < 60 years old	Elderly > 75 years old
Total number of hips	261 (229 patients)	139 (131 patients)
Gender		
Men	150 (57.5)	63 (45.3)
Women	111 (42.5)	76 (54.7)
Age at surgery (years)	52.1 (24.3–59.9)	79.1 (75.0–91.3)
Indication for surgery		
Primary osteoarthritis	166 (63.6)	112 (80.6)
Secondary osteoarthritis	27 (10.3)	18 (12.9)
Inflammatory arthritis	4 (1.5)	2 (1.4)
Avascular necrosis of the femoral head	24 (9.2)	5 (3.6)
Fracture	0 (0.0)	1 (0.7)
Congenital dysplasia of the hip	40 (15.3)	1 (0.7)
Surgical approach		
Direct anterior	42 (16.1)	20 (14.4)
Anterolateral	211 (80.8)	117 (84.2)
Direct lateral	6 (2.3)	1 (0.7)
Posterolateral	1 (0.4)	0 (0.0)
Transgluteal with GT osteotomy	1 (0.4)	1 (0.7)

N (%)

Preoperatively femur morphology was assessed for all patients using the Dorr classification [22].

**Fig. 1:**
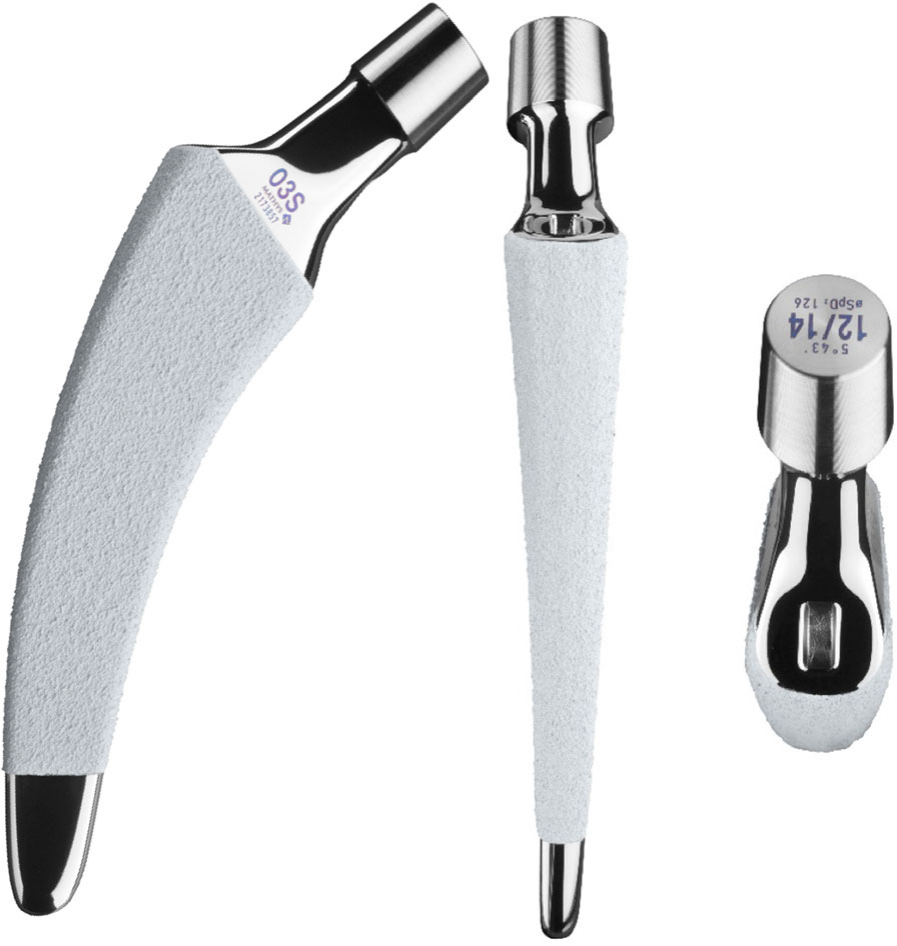
The optimys short stem (Mathys Ltd., Bettlach, Switzerland)

In all patients, a cementless calcar-guided short stem (optimys, Mathys Ltd. Bettlach, Switzerland), which is available in 12 different sizes with a 12/14 mm taper and 2 different offset versions, was implanted (Fig. 1). A rough titanium plasma sprayed coating, and a calcium phosphate coating are provided to ensure safe metaphyseal anchorage in the femoral bone. The stem is aligned along the proximal medial cortex and the calcar femorale. Anchoring is based on the fit-and-fill principle and can be done as classic 3-point anchoring [7].

The optimys stem was mainly combined with cementless press-fit cups (RM Pressfit vitamys, Mathys Ltd. Bettlach, Switzerland; Fitmore and Allofit, Zimmer, Warsaw, IN, USA) using either a ceramic-polyethylene or ceramic-ceramic bearing couple. The surgical approach was dependent upon surgeon preference (Tab. 1). All patients received perioperative doses of prophylactic antibiotics. Full weight bearing was generally allowed in all cases immediately after surgery except for those cases an intraoperative complication, such as a fissure, was detected.

Clinical follow-up included assessment of the Harris Hip Score (HHS) and the Visual Analogue Scale (VAS) for pain at rest and on load as well as overall patient satisfaction.

Radiological follow-up was assessed using a standardized and calibrated AP view pelvis radiograph. Using a modification of the zones described by Gruen [23], bone resorption, cortical hypertrophy, osteolysis, and radiolucency were analyzed at last follow-up on the standardized radiograph according to Kutzner et al. [24]. To detect bone resorption and osteolysis, the proximal femoral bone was scanned in order to find areas with enhanced bone transparency and thinned or resorbed trabeculae according to the Singh-Index [25]. Grades 1–3 were considered to be bone resorption. Cortical bone width was measured preoperatively and during follow-up to detect any variation. Radiolucent lines were detected, and the maximal width was measured.

Patient demographics and surgery related intraoperative and postoperative complications, including peri- and postoperative periprosthetic fractures, as well as stem revisions were documented.

For statistical analysis, the SAS Enterprise Guide Version 7.11 (SAS Institute, Cary, North Carolina, USA) was used. A Wilcoxon signed-rank test was used to compare continuous variables between groups and the Fisher exact test was used to analyze contingency tables. Log rank test was used to detect different survival rates. A *p* value of < 0.05 was defined as significant.

## Results

**Fig. 2:**
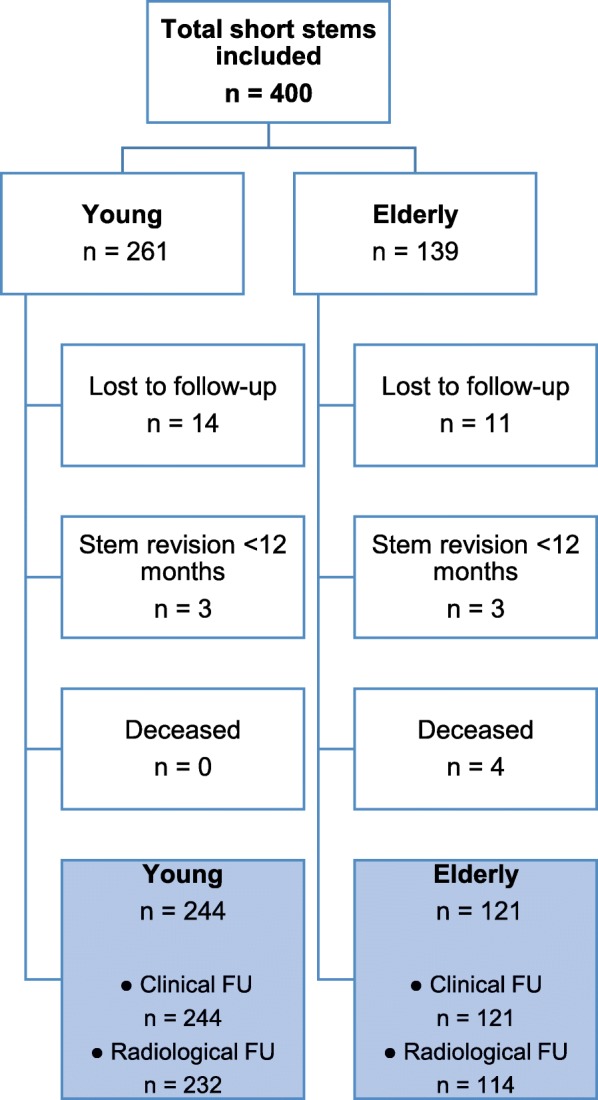
Study Flowchart

We identified 14 hips lost-to-follow-up in the young group and 11 in the elderly group. Four elderly patients were known to be deceased before the minimum follow-up of 12 months and 6 patients, 3 from each group, underwent stem revision for various reasons before the time point of 12 months of follow-up. Thus, for clinical follow-up, the young group (< 60 years) consisted of 244 hips in 214 patients with a mean age of 52.1 years (SD 6.7) and a mean follow-up of 52.0 months (SD 17.9; range 12.6–81.2 months) and the elderly group (> 75 years) consisted of 121 hips in 114 patients with a mean age of 79.1 years (SD 3.6) and a mean follow-up of 43.3 months (SD 21.3; range 12.0–75.5 months) (Fig. 2).

Regarding preoperative Dorr types, significant differences were found comparing the two groups. In the young group most femurs were classified Dorr type A and there was only one Dorr type C femur, whereas in the elderly group most femurs were classified Dorr type B and 5.8% of the cases were found to be Dorr type C (Table 2).

**Table 2:** Distribution of preoperative Dorr types

	Young < 60 years old	Elderly > 75 years old	*p*-value
Preoperative Dorr type			
A	169 (64.8)	27 (19.4)	< 0.0001
B	91 (34.9)	104 (74.8)	
C	1 (0.4)	8 (5.8)	
Total	261 (100.0)	139 (100.0)	

N (%)

HHS at last follow-up improved significantly for both groups compared to the mean values before surgery. The difference of HHS between the two groups was found to be statistically significant (*p* < 0.0001) for both preoperative and postoperative values. No differences were found for the mean VAS values of rest pain, load pain and satisfaction between the two groups. The clinical outcome is summarised in Table 3.

**Table 3:** Clinical and radiological outcome at last follow up

	Young < 60 years old	Elderly > 75 years old	*p*-value
Clinical outcome			
Harris Hip Score			
Preoperative	47.6 (16.1)	40.9 (16.8)	*p* < 0.0001
Last follow-up	96.8 (7.8)	91.0 (13.1)	*p* < 0.0001
VAS rest pain			
Preoperative	4.7 (2.5)	4.5 (2.7)	*p* = 0.28
Last follow-up	0.2 (0.9)	0.1 (0.6)	*p* = 0.77
VAS load pain			
Preoperative	7.5 (1.9)	7.7 (1.8)	*p* = 0.14
Last follow-up	0.6 (1.2)	0.5 (1.3)	*p* = 0.41
VAS satisfaction			
Preoperative	2.5 (2.3)	2.9 (2.5)	*p* = 0.07
Last follow-up	9.6 (1.0)	9.4 (1.4)	*p* = 0.56
Radiological outcome			
Bone resorption			
No	197 (84.9)	99 (86.8)	*p* = 0.75
Yes	35 (15.1)	15 (13.2)	
Cortical hypertrophy			
No	220 (94.8)	110 (96.5)	*p* = 0.59
Yes	12 (5.2)	4 (3.5)	
Osteolysis			
No	231 (99.6)	114 (100.0)	*p* = 1.0
Yes	1 (0.4)	0 (0.0)	
Radiolucency			
No	228 (98.3)	112 (98.2)	*p* = 1.0
Yes	4 (1.7)	2 (1.8)	

Mean (standard deviation) for clinical outcome,; VAS: Visual analogue scale; N (%) for radiological outcome; clinical outcome based on 244 young hips and 121 elderly hips; radiological outcome based on 232 young hips and 114 elderly hips

For both groups, resorption of the proximal femoral bone was mainly detected in Gruen-zones 1, 2 and 7. Signs of osteolysis could only be identified in one hip in the young group in Gruen-zone 1 without any clinical consequences. Cortical hypertrophy could be found almost exclusively in Gruen-zones 3 and 5. Radiolucent lines of less than 2 mm were detected in Gruen-zones 1 and 2 in 1.7 and 1.8% of the hips, respectively. Comparing the young group and the elderly group, none of the radiological parameters that have been assessed reached differences of statistical significance. The results of the radiological analysis are shown in Table 3.

Intraoperative and postoperative complications, as well as rates and reasons for stem revision, were assessed in both groups (Table 4). Postoperative local complications, such as wound dehiscence, hematoma/seroma, superficial infection and nerve palsy were documented in the young group in 4.2% and in the elderly group in 5.7%, respectively. Two dislocations occurred in the young group during rehabilitation. One was treated conservatively, and in the other case, the acetabular component was revised. In one patient in the young group the ceramic head fractured 3 years postoperatively leading to revision surgery with retention of the stem.

**Table 4:** Complications

		Young < 60 years old	Elderly > 75 years old	*p*-value
Intraoperative complications				
Femur fissure		4 (1.5)	2 (1.4)	*p* = 1.0
Avulsion of trochanter		1 (0.4)	1 (0.7)	*p* = 1.0
Postoperative complications				
Wound dehiscence		1 (0.4)	0 (0.0)	*p* = 1.0
Nerve palsy		4 (1.5)	2 (1.4)	*p* = 1.0
Superficial infection		2 (0.8)	2 (1.4)	*p* = 0.61
Wound healing disorder		2 (0.8)	0 (0.0)	*p* = 0.55
Haematoma / seroma		8 (3.1)	9 (6.5)	*p* = 0.12
Dislocation		2 (0.8)	0 (0.0)	*p* = 0.55
Fracture ceramic head		1 (0.4)	0 (0.0)	*p* = 1.0
Periprosthetic fracture	Vancouver type A	0 (0.4)	1 (0.0)	
	Vancouver type B	0 (0.0)	3 (2.1)	
	Vancouver type C	1 (0.4)	1 (0.7)	
	Total	1 (0.4)	5 (3.6)	*p* = 0.02

N (%)

Intraoperative periprosthetic femoral fractures occurred in 1.5% in the young group and in 1.4% in the elderly group (*p* = 1.0). In 6 cases an intraoperative femoral fissure was observed, and in 2 cases an avulsion fracture of the greater trochanter occurred, mainly treated conservatively with mobilization without full weight bearing and occasionally operatively with either cerclage wiring or the selection of a different implant.

**Fig. 3:**
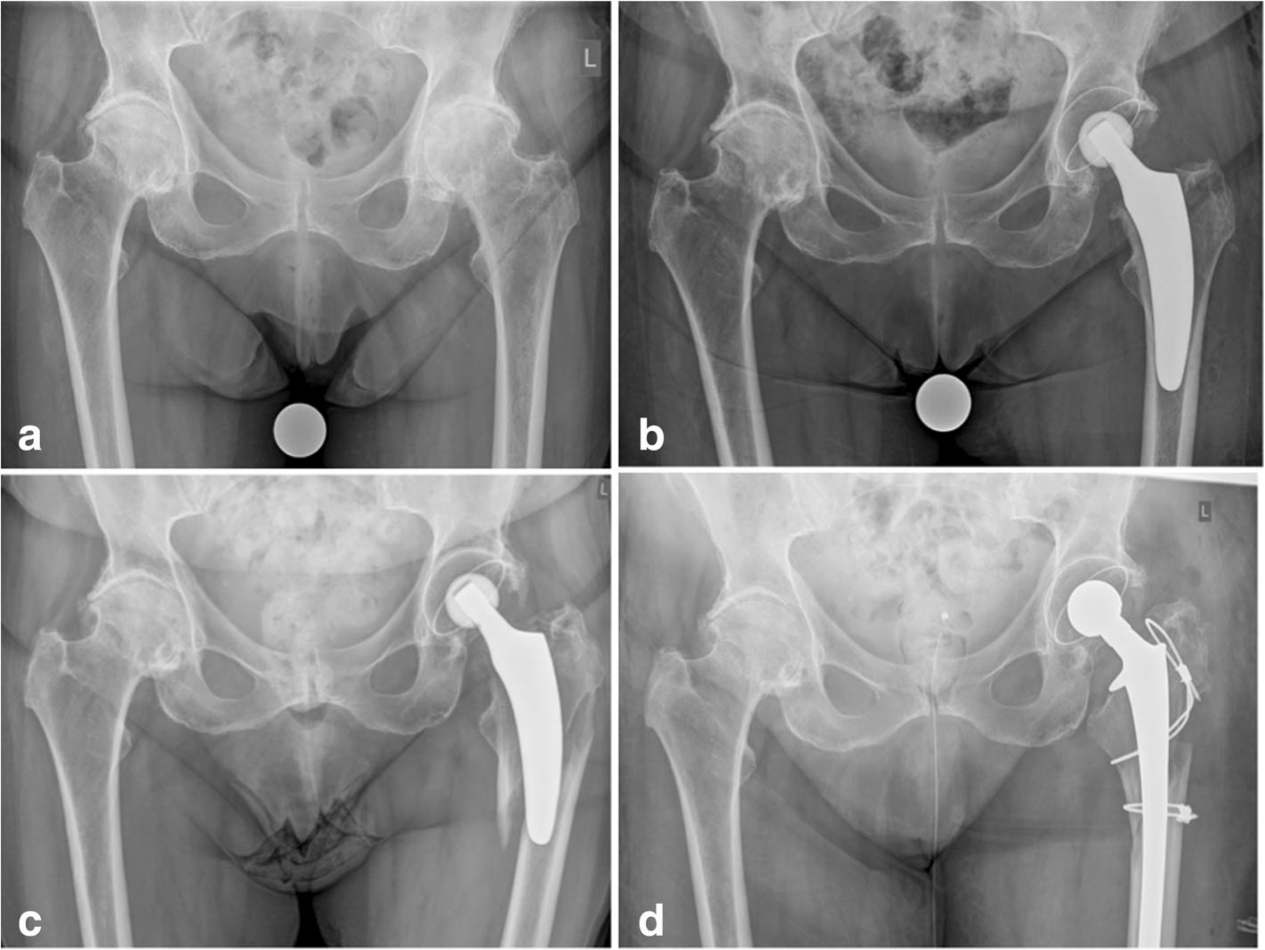
Example of postoperative periprosthetic fracture Vancouver type B3 due to accidental fall of an elderly female patient followed by stem revision (**a**: preoperative; **b**: postoperative; **c**: periprosthetic fracture; **d**: after stem revision)

**Fig. 4:**
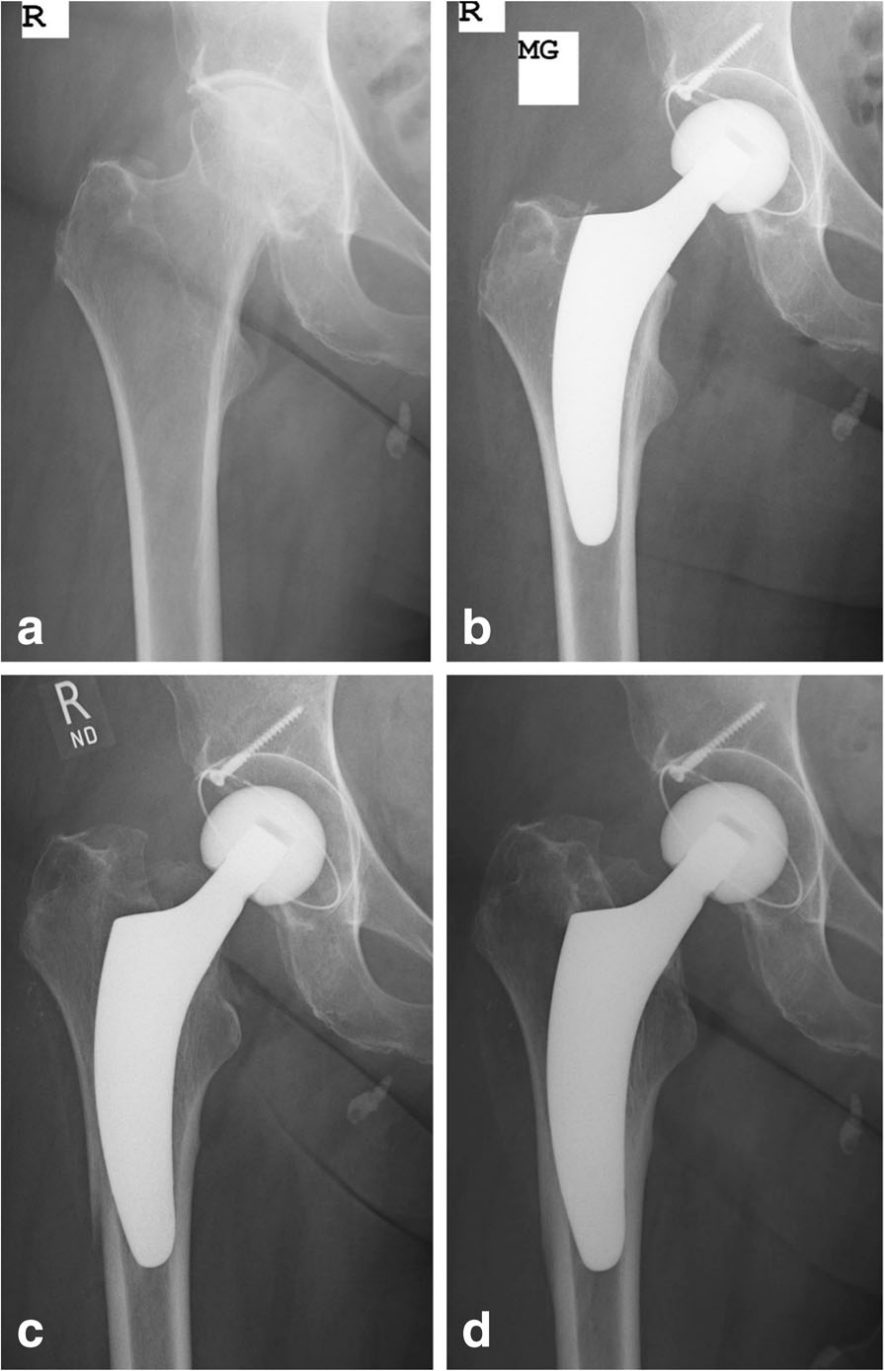
Example of postoperative periprosthetic fracture Vancouver type B in the course of severe subsidence in an elderly female patient treated conservatively (**a**: preoperative; **b**: postoperative; **c**: periprosthetic fracture with severe subsidence; **d**: follow-up after 24 months)

Postoperative periprosthetic femoral fractures during follow-up were observed in 0.4% in the young group and 3.6% in the elderly group respectively, the difference being statistically significant (*p* = 0.02). Three of these periprosthetic fractures led to stem revision and the other 3 were successfully treated conservatively with no weight bearing using crutches (Figs. 3 and 4). Whereas in Dorr type A femurs no postoperative periprosthetic fracture was observed at all, in type B femurs a total of 4 fractures occurred and in type C femurs a total of 2 fractures occurred respectively. Thus, the incidence of postoperative periprosthetic fractures in Dorr type B and Dorr type C femurs was 2.1 and 22.2%, respectively (*p* = 0.0001) (Table 5).

**Table 5:** Analysis of postoperative periprosthetic fractures regarding different Dorr types

	Perisprosthetic fracture (*n* = 6)	No periprosthetic fracture (*n* = 394)	*p*-value
Dorr type			
A	0 (0.0)	196 (100.0)	< 0.0001
B	4 (2.1)	191 (98.1)	
C	2 (22.2)	7 (77.8)	

N(%)

**Fig. 5:**
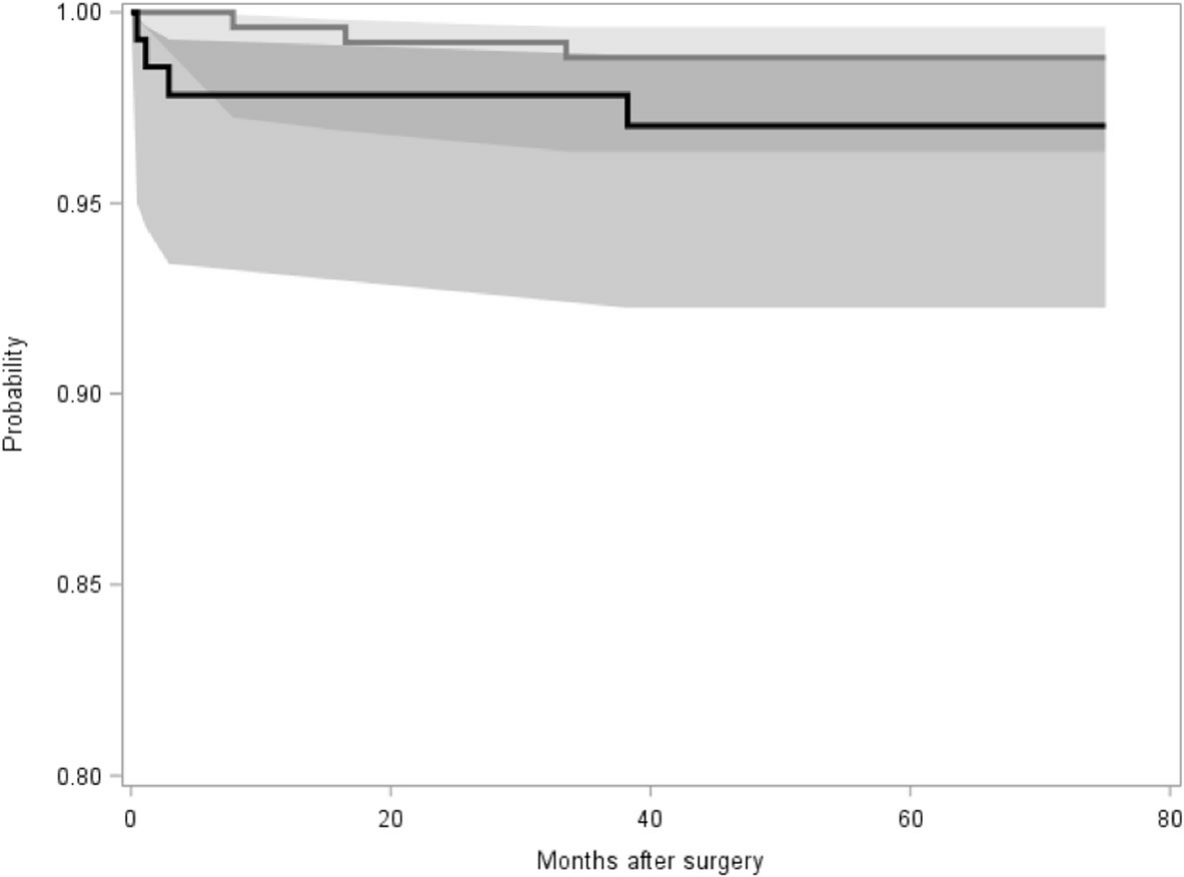
Kaplan-Meier survival curve for the young group (grey) and the elderly group (black). Log rank test revealed no significant difference between the groups (*p* = 0.1994)

Additionally, all other reasons for stem revision were documented (Table 6). A total of 7 stem revisions had to be performed during follow-up, besides the femoral periprosthetic fractures mainly due to aseptic loosening (3 cases) and infection (1 case). In the young group, stem revision rate was 1.1%, whereas in the elderly group, 2.8% of all stems had to be revised during follow-up (*p* = 0.24). Survival rate (endpoint stem revision for any reason) was calculated at 60 months to be 98.81% for the young group and 97.02% for the elderly group. Log rank test revealed no significant difference between the groups (*p* = 0.1994) (Fig. 5).

**Table 6:** Reasons and rates for stem revision

	Young < 60 years old	Elderly > 75 years old	*p*-value
Total	3 (1.1)	4 (2.8)	*p* = 0.24
Aseptic loosening	3 (1.1)	0 (0.0)	*p* = 0.55
Periprosthetic fracture	0 (0.0)	3 (2.2)	*p* = 0.04
Deep infection	0 (0.0)	1 (0.7)	*p* = 0.35

N (%)

## Discussion

The aim of this study was to investigate complications and the clinical and radiological outcome comparing two distinct age groups that received the same cementless calcar-guided short stem. We asked the question if this particular short-stem design, which to date still is mainly used in young and active patients, can be implanted safely in elderly patients. To our knowledge, it is the first study of its kind as far as this short-stem design is concerned.

When it comes to elderly patients, lower revision rates for cemented THA compared to cementless THA have been documented from registry outcomes worldwide [26, 27]. Nevertheless, there is a tendency, in some parts of the world, to also treat the elderly using cementless implants. This phenomenon has been called, the “Uncemented Paradox” by Troelsen et al. [28]. Cemented THA is associated with an increased incidence of cardiovascular adverse events [29]. The bone cement implantation syndrome (BCIS) is a well-known and potentially fatal complication [30]. The morbidity and mortality, associated with these cement-related complications are by definition not an issue with cementless THA and lower postoperative mortality rates have been reported for cementless THA [31].

On the one hand there are some potential benefits that cementless short-stem THA can offer to geriatric populations. Elderly patients undergoing THA are usually frail and with significant comorbidities. For this reason, they can particularly benefit from approaches or implants that are less invasive and thus associated with less intraoperative blood loss and postoperative transfusions, which can interfere with early mobilization after THA. Hochreiter et al. [16] recently found that the investigated short stem was associated with less blood loss and blood transfusion compared to a standard cementless straight stem. The less invasive operative technique in calcar-guided short-stem THA, sparing bone and soft tissue by allowing a “round-the-corner” insertion, also leads to short operation times [17]. However, a distinct learning curve with these short-stem designs for surgeons new to this technique has to be taken into account [32]. Additionally, these stem designs have been shown to successfully reconstruct patients’ anatomy by providing favorable early clinical results [33, 34].

This can be confirmed by the present study. The early clinical outcome of short-stem THA in the elderly group is encouraging. No differences were found for the mean VAS values of rest pain, load pain, and satisfaction between the two groups. The difference of HHS preoperatively and at last follow-up is somewhat expected and can be explained by the fact that elderly patients usually have more comorbidities that affect normal gait and are generally less active than younger ones. The differences in these two parameters influence greatly the calculation of this specific score [35]. Previous studies comparing young versus elderly collectives showed similar differences in postoperative HHS [19].

On the other hand, various questions arise, if, given the shortened design with less diaphyseal anchorage, a safe bony ingrowth/ongrowth can be accomplished particularly in elderly patients with reduced bone quality and if stress-shielding, as well as stress-rising, can be avoided [36].

Resorption of femoral bone and cortical hypertrophy were slightly less for the elderly group, the difference not being statistically significant. In 1.8% versus 1.7% of the cases radiolucent lines were observed and in only one case of the young group signs of osteolysis were seen. Thus, regarding radiological alterations, no disadvantages were detected in the elderly group during short-term follow-up.

Our results match those reported in the literature. In a recent publication by Boller et al. no significant differences or any influences of osteointegration and clinical outcome of the cementless Metha short stem (BBraun, Aesculap, Tuttlingen, Germany) for young (< 60 years) and elderly (> 60 years) patients were detectable [12]. They concluded that short stems are to be considered adequate treatment also in geriatric patients. Kim et al. [21] compared two groups of elderly and younger patients and found that an ultra-short cementless stem (Proxima DePuy, Warsaw, IN, USA) with a metaphyseal flare can achieve stable primary fixation with a similar clinical outcome. In a different investigation with a mean follow-up of 4.6 years, Kim et al. [37] found strong evidence that THA using the same short cementless metaphyseal fitting stem in patients aged 70 years and older provides satisfying clinical results. However, it must be outlined that the design philosophy of the investigated short stem was significantly different from the one used in the present study. In a retrospective study by Yu et al. [38], a cementless short stem (Tri-Lock, DePuy, Warsaw, In, USA) was compared to a cementless standard stem in patients over 70 years old with no statistically significant clinical and radiological differences between the two groups, with the exception of the incidence of thigh pain which was significantly higher in the conventional stem group. Morales de Cano et al. [39] in a recent investigation shared their experience with the GTS conservative short stem (Biomet, Warsaw, IN, USA) in two groups of patients, younger and older than 70 years, and also found no difference regarding clinical and radiological outcomes, supporting the safe usage in elderly patients.

The potential interference with osseointegration of cementless short stems in geriatric patients with symptoms of osteoporosis might also increase the risk of aseptic loosening [19]. In a recent “Einzelbild-Roentgen-Analysis” (EBRA-FCA) investigating early axial stem migration age had no influence on the outcome using the investigated short stem [40]. In this cohort, no revision had to be performed after 2 years. In the present study, stem revision due to aseptic loosening had to be performed in three cases during follow-up. However, these cases were observed exclusively in the young group, whereas no loosening was noticeable for the elderly group. This can be explained by an increased activity level, as seen in younger patients and those with unrestricted mobility, being an important factor in the etiology of loosening [41]. THA in young patients has been historically associated with lower survivorship, mainly due to aseptic loosening [42].

Another issue that raises concerns about safely using cementless short stems in the elderly is the risk of periprosthetic femoral fractures [43, 44]. Intraoperative fissures were 1.5% in the young group versus 1.4% in the elderly group, thus, no increase in risk for geriatric patients was remarkable. Given the rounded and shortened design of calcar-guided short stems, avulsion-fractures of the greater trochanter can be reduced significantly compared to trochanter violating stem designs. Local registry data published by Mai et al. [45] reported a fracture rate of 0.3% for the investigated short stem compared to 4% for a Spotorno type stem, also indicating low risks for elderly patients. The above-mentioned study by Yu et al. [38], investigating the Tri-Lock stem (Depuy, Warsaw, IN, USA), found that no intraoperative periprosthetic fracture occurred with the short stem, whereas for the conventional stem an intraoperative fracture rate of 8.6% was observed. In a recent investigation of Kim et al., comparing a cementless short stem with cemented conventional stems in octogenarians, for both types of implants a rigid fixation could be obtained. However, the incidence of undisplaced periprosthetic calcar fractures intraoperatively was significantly higher in the cemented stem group [13].

These results strongly support the intraoperative advantage of short stems in elderly patients intraoperatively. However, in the present study, a significantly higher number of postoperative periprosthetic fractures were observed in the elderly group during follow-up compared to the group of young patients. A total of 3 stem revisions had to be performed due to postoperative femoral fractures, all of which occurred in the elderly group. This potentially may reflect an increase of risk for elderly patients regarding the usage of calcar-guided short-stem THA. In a recent publication regarding periprosthetic femoral fractures as cause of early revision following short-stem THA, Kim et al. concluded that advanced age, Dorr type C femoral morphology and the use of calcar-loading, metaphyseal anchoring stems increased the risk of periprosthetic fractures [46]. However, all kinds of different stem-designs were used. Also Gromov et al. found Dorr type C femurs to be an independent risk factor for early periprosthetic fractures using a double tapered cementless stem [47]. At the same time, comparing Dorr types A, B and C following implantation of the Proxima short stem (Depuy, Leeds, UK), Kim et al. investigated no significant differences regarding implant survival at mid-term [48]. These results lead to the conclusion, that an increased risk of early periprosthetic fractures in Dorr type C femurs might be highly dependent on implant-design. In the present investigation the incidence of fractures in those femurs that have been classified Dorr type C was 22.2%, which implies that surgeons have to be very cautious regarding the indication in markedly reduced bone quality, given the investigated stem-design. However the range of age in those patients with postoperative periprosthetic fractures is quite high (53.8–80.4 years) not being restricted to only elderly patients. Mean age of all elderly patients included was found to be 79.1 years (range 75.0–91.3 years) whereas mean age in those patients with fracture was 73.8 years. Thus, not necessarily advanced age alone but particularly reduced bone quality and Dorr type C femur morphology is associated with an increased risk of postoperative periprosthetic fractures. It remains unknown if these fractures can also partially be explained simply with the higher risk of accidental fall in the elderly and might not necessarily reflect the risk of the type of implant chosen. Almost all postoperative fractures in the present study occurred following an accidental fall mainly during rehabilitation. Although a lower risk of cemented THA regarding postoperative periprosthetic fractures has been described by several studies, it remains unknown, if cemented THA in the present cases would have prevented these fractures from happening [49, 50]. In one case in the elderly group, a Vancouver type B fracture occurred after 2 months in the course of a severe axial subsidence. The positioning and sizing of the stem, in this case, markedly increasing femoral offset, however, must be considered a surgical mistake (Fig. 4). Conservative treatment with no weight bearing led to fracture healing and a satisfied patient. Overall, regarding the present results a learning curve has to be acknowledged, since all patients included to the study were the first ones with the investigated stem being used, coming from each of the five centres.

To the present study, limitations must be acknowledged. Most importantly the present study lacks a randomized controlled design. Consequently, findings have to be interpreted with caution and further investigations are mandatory. Second to be mentioned is the short-term follow-up. Although only long-term results should be considered valid, initial evaluation of bony alterations and complications is obligatory to identify undesirable outcomes [51]. Third, the radiological method used to evaluate femoral bone loss and stress-shielding is rather inaccurate compared to dual-energy x-ray absorptiometry (DEXA) scans. However, given the size of the collective, this method would have resulted in intense effort and costs. Future investigations using more accurate methods are necessary in order to validate these results.

As a further limitation, there is a marked difference in the size of the two investigated age groups. This can be mainly explained by the above-mentioned registry-backed tendency to treat older patients with cemented stems because of poor bone quality.

Finally, since the study investigated only one design of short stem, the present results cannot be generalized to different cementless short-stem designs.

## Conclusions

Advanced age alone is not necessarily to be considered as contra-indications for calcar-guided short-stem THA, although further follow-up is needed. Except for the Harris Hip Score, no statistically significant differences in the clinical and radiological outcome between the two groups of young and geriatric patients were detected.

During follow-up, however, a tendency towards a higher probability of revision surgery in the elderly can be observed, although rates of stem revision did not differ statistically between young and geriatric patients. While in young patients aseptic loosening is the main cause for implant failure, incidence of periprosthetic fractures in the group of elderly patients was significantly increased, potentially leading to revision surgery. Markedly reduced bone quality with femur morphology of Dorr type C seems to be associated with an increased risk for postoperative periprosthetic fractures, thus indication should be limited to Dorr types A and B.

Further follow up is necessary in order to draw safe conclusions about the long-term behavior of these implants in the elderly.

## Abbreviations

BCIS: Bone Cement implantation syndrome
DEXA: Dual-Energy Xray Absorptiometry
EBRA-FCA: “Einzel Bild Roentgen Analysis”-Femoral Component Analysis
HHS: Harris Hip Score
SD: Standard Deviation
THA: Total hip arthroplasty
VAS: Visual analogue scale
